# Whole-Brain Confocal Imaging Provides an Accurate Global View of the Nigral Dopamine System

**DOI:** 10.3390/diagnostics15111436

**Published:** 2025-06-05

**Authors:** Fu-Ming Zhou

**Affiliations:** Department of Pharmacology, Addiction Science and Toxicology, College of Medicine, University of Tennessee Health Science Center, Memphis, TN 38163, USA; fzhou3@uthsc.edu

**Keywords:** basal ganglia, cerebral cortex, confocal imaging, dopamine, striatum, Parkinson’s disease, schizophrenia

## Abstract

Clinicopathological studies and the effectiveness of dopaminergic replacement therapy establish that dopamine loss is the key pathology causing motor symptoms in Parkinson’s disease. The dopamine neurons that are impaired in Parkinson’s disease reside in the substantia nigra and ventral tegmental area in the midbrain. These neurons project into the striatum, where dopamine axons bifurcate repeatedly and form dense axon networks (the striatum is separated into the caudate nucleus and putamen by the internal capsule). Midbrain dopamine neurons also innervate many other areas of the brain, including the cerebral cortex. Therefore, there are preclinical and clinical studies investigating extrastriatal dopamine mechanisms in motor control and Parkinson’s disease pathophysiology and treatment. While extrastriatal dopamine can contribute, this contribution needs to be compared with the contribution of the striatal dopamine system. An isolated view of the extrastriatal dopamine system is like examining only the ear of an elephant and may lead to distorted assessments for preclinical and clinical research and diagnostic work. Thus, photographs of the whole brain dopamine system are important. For these reasons, we photographed the dopamine systems in whole mouse brain sagittal sections, showing clearly that, under identical imaging conditions, dopamine innervation is highly concentrated and intense in the striatum but sparse and weak in the cerebral cortex.

**Figure 1:**
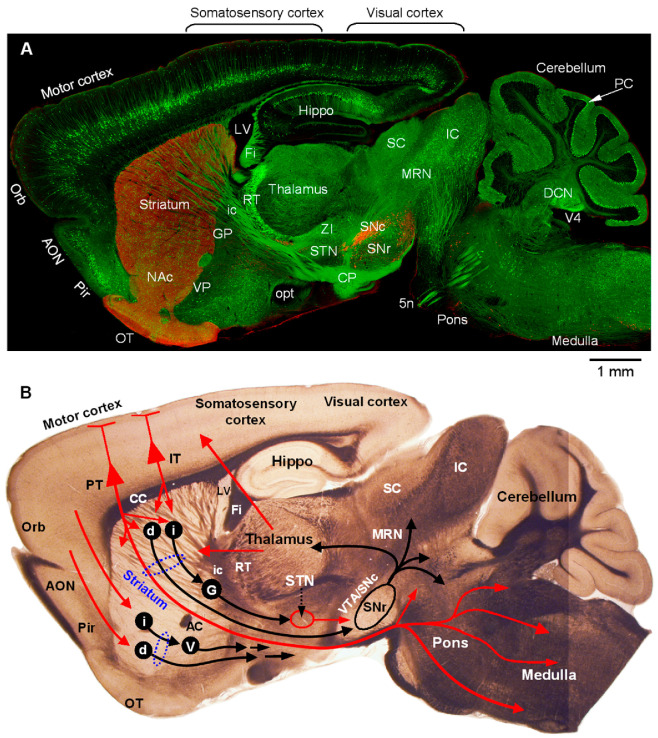
Whole-brain confocal imaging provides a global view of the dopamine system and the cortico-basal ganglia–thalamic circuit. The top panel (**A**) is a high-resolution confocal picture of a whole sagittal brain section of a 2-month-old male mouse. Subsets of cortical pyramidal neurons (mostly in layer 5) and their axons are labeled by Thy1-YFP. Subsets of GABAergic neurons in the brain, including cerebellar Purkinje cells, are labeled by GAD1-eGFP and GAD2-eGFP. The tissue section was also immunostained for tyrosine hydroxylase (TH), the key enzyme for dopamine synthesis. YFP and GFP were detected using a 488 nm excitation laser and TH-red secondary antibody was detected using a 561 nm excitation laser on a laser-scanning confocal microscope with a 20× NA 0.8 objective. The Z-stacking, tiling, scanning, and stitching functions of the imaging system were used for obtaining high-resolution images of large tissue sections. The entire brain section was photographed under identical imaging conditions such that the different immunostaining signal intensities indicate true differences in dopamine innervation. Key information flow directions are provided in the separate bright field picture (**B**) of a fresh brain section at a similar anatomical position. These unique and high-quality imaging results, together with Figure 2, provide a clear, easy-to-understand, and compelling visualization of the nigro-forebrain dopamine system.

**Figure 2:**
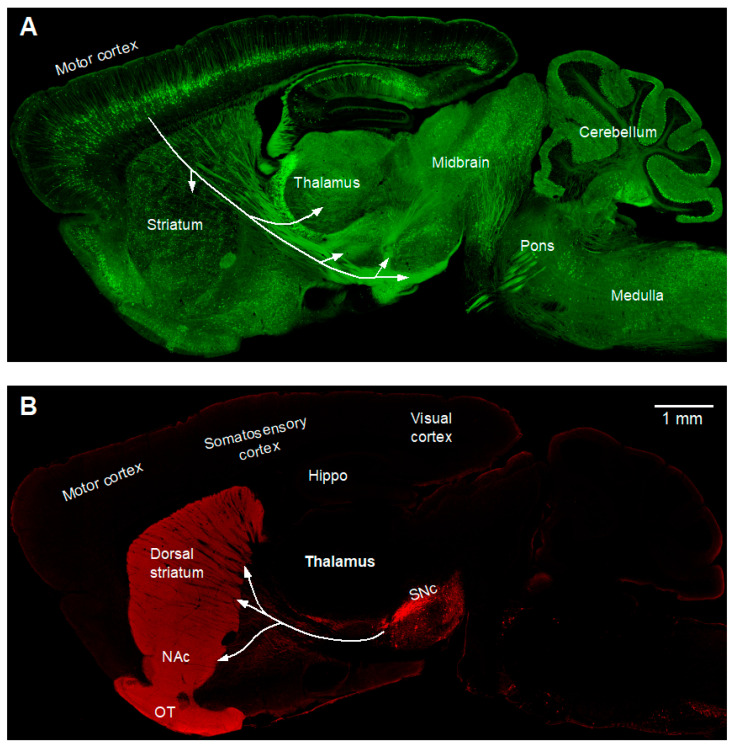
The green and red channels of the picture in Figure 1A are displayed separately to show more details of brain structures. (**A**). YFP-labeled cortical neurons illustrate corticofugal axons going to the striatum, the subthalamic nucleus, other basal ganglian nuclei, thalamus, and also the brainstem and spinal cord. Subsets of GABA neurons, including cerebellar Purkinje cells, are labeled with GFP. In this experiment, we used the same 488 nm laser for detecting YFP and GFP such that the YFP neurons and processes and GFP neurons and processes were not separated and viewed as green, although anatomical locations separate them to some degree. (**B**). Under identical staining and imaging conditions for all brain areas in the section, TH signal (red) is very strong in the striatum but weak/barely visible in the cerebral cortex. Because the staining and imaging conditions were identical, these results reveal the true, highly concentrated DA innervation in the striatum and sparse dopamine innervation in the cerebral cortex. It is important to note that if we stain and/or image cortical and striatal TH separately and image cortical TH to achieve maximal intensity (a natural and common practice when the research is focused on the cortical dopamine system and the scientist needs to save time and user fees for imaging equipment), cortical TH/dopamine innervation will be substantially higher than that shown here, but this is unintentionally taking the result out of its context and is therefore misleading. The corticobrainstem projection and corticospinal projection are clearly essential for motor function while the cortico-basal ganglia–thalamo–cortical loop is required for motor function and cognition [1,2,3,4]. Nigrostriatal dopamine projection is clearly needed for normal brain cognitive and motor functions, as indicated by Parkinson’s disease (caused by a severe degeneration of nigral dopamine neurons and their axonal projection to the striatum) and schizophrenia (its key symptoms are associated with dopaminergic overactivity) [5,6,7]. Abbreviations used here and in Figure 1 are listed at the end of the text.

**Figure 3:**
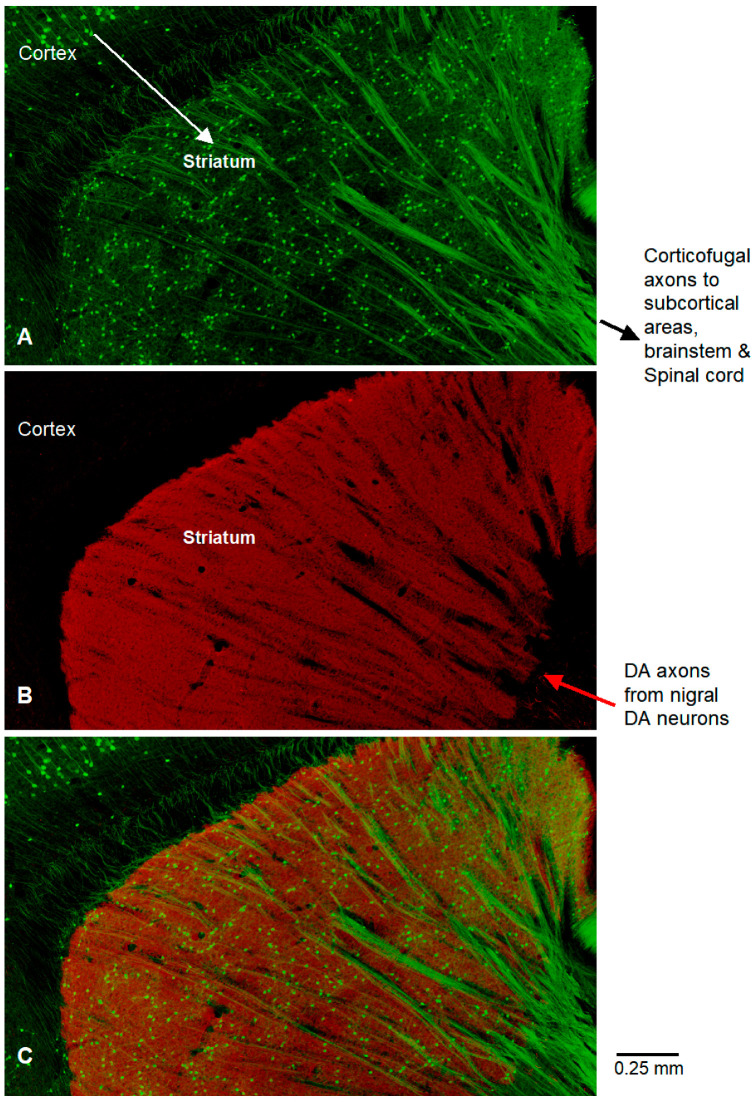
These three zoomed-in pictures of the dorsal striatal area of Figure 1A highlight the intense TH-labeled dopamine innervation (red, (**B**)) in the dorsal striatum and the strikingly sharp difference between striatal and cortical dopamine innervation. Notice YFP-labeled corticofugal axons (green, (**A**)) going through dorsal striatum ((**C**), merged image of (**A**,**B**)). Also notice that these corticofugal axons often form bundles (**A**,**C**), maybe because, during development, they were trying to reach the same destinations and traveling together on pioneer-proven trails to help them navigate, like travelers traveling together in a forest when their destinations are in the same direction. The green neurons in the striatum in (**A**,**C**) are the medium spiny neurons labeled by GAD-GFP (these neurons appear greenish due to overlay red signal). Intense dopaminergic activity regulates the intrinsic excitability and synaptic inputs and outputs, and thus regulates brain functions [6,8,9,10,11,12,13,14]. Our photographic data show a profound concentration of dopaminergic axon innervation in the striatum among different brain areas. This is consistent with quantitative neurochemical studies. HPLC measurements in brain regions show a striking concentration of dopamine in the striatum: the striatal dopamine tissue content vs. frontal cortical dopamine content ratio was ~70:1 in rodents and humans [15,16,17,18]. The photographic data in this paper are important and useful for the following reasons. Our single photograph capturing the brain’s main dopamine system, the nigroforebrain dopamine system, in a sagittal mouse brain section, with pyramidal neurons and subsets of GABA neurons, at the high resolution of a 20× objective, is unique and informative. The sagittal brain section contained the substantia nigra, striatum, thalamus, motor and somatosensory cortices, and several other key brain structures. Thus, although not covering the whole brain, this picture provides a reasonably accurate and clear visualization of the nigro-forebrain dopamine system for the whole brain. Because the entire brain section is captured in a single photograph, the imaging conditions were identical for all the different brain areas and structures in the sagittal brain section; consequently, the different dopamine signal intensities in different brain areas captured in this picture are real differences in dopamine innervation. This is important because the captured dopamine signal intensity can be strongly affected by imaging conditions. For example, if the cortical dopamine system is the target of a study, the scientist will focus on the cerebral cortex and optimize the imaging conditions to photograph dopamine axons (although these imaging conditions will saturate the dopamine signal in the striatum). This focused approach is suitable for some experimental questions, such as determining cortical dopamine innervation, but it can confound and distort functional studies on the cortical dopamine system; indeed, functional studies on the cortical dopamine system reported highly conflicting results (dopamine both excited and inhibited cortical pyramidal neurons), therefore, it was concluded that dopamine’s effect is neither excitatory nor inhibitory [19]. However, for dopamine to affect neuronal activity, excitation and inhibition are the only two possible effects; the third possibility is that the cortical dopamine system is weak and its effects on cortical pyramidal neuron activity are too small to be reliably detected, leading to conflicting and potentially unreliable experimental results reported in the literature. In conclusion, our photographic data provide an accurate global visualization of the nigro-forebrain dopamine system, and this accurate information is important and useful for understanding the pathophysiology and neuronal circuitry dysfunction of Parkinson’s disease and schizophrenia.

## Data Availability

All data are included in the pictures in this paper.

## Abbreviations

5n: trigeminal nerve; AC: anterior commissure; AON: anterior olfactory nucleus; CC: corpus callosum; CNS: central nervous system; DNC, deep cerebellar nuclei; Fim, fimbria; GABA: γ-amino butyric acid; G: GPe: globus pallidus external segment; GPi: globus pallidus internal segment; GAD1 and GAD2: glutamic acid decarboxylase 1 and 2; ic, internal capsule; IC: inferior colliculus; SC: superior colliculus; IT: intratelencephalic pyramidal neurons; PT: pyramidal tract neurons; V: ventral pallidus; d: medium spiny neuron in the direct pathway expressing D1-type dopamine receptor; i: medium spiny neuron in the indirect pathway expressing D2-type dopamine receptor; IC: internal capsule; LC: locus coeruleus; LO: lateral olfactory tract; LV: lateral ventricle; MCPO: magnocellular preoptic nucleus; MRN: midbrain reticular nucleus; NAc: nucleus accumbens; NE, norepinephrine, Orb: orbital front cortex; PC, Purkinje cell, PFC: prefrontal cortex; SNc: substantia nigra pars compacta; SNr: substantia nigra pars reticulata; STN: subthalamic nucleus; TH: tyrosine hydroxylase; V4, fourth ventricle; VTA: ventral tegmental area; ZI: zona incerta.
